# Die Luft, die wir alle atmen: Zur Arbeit der Kommission Innenraumlufthygiene (IRK)

**DOI:** 10.1007/s00103-023-03815-z

**Published:** 2024-01-09

**Authors:** Wolfram Birmili, Anja Daniels, Ana Maria Scutaru, Frank Kuebart

**Affiliations:** 1 Geschäftsstelle Kommission Innenraumlufthygiene (IRK), Fachgebiet II 1.3 „Innenraumhygiene und gesundheitsbezogene Umweltbelastungen“, Umweltbundesamt, Corrensplatz 1, 14195 Berlin, Deutschland; 2 Vorsitz Kommission Innenraumlufthygiene (IRK), eco-Institut Germany GmbH, Schanzenstr. 6–20, 51063 Köln, Deutschland

Liebe Leserinnen und Leser,

im Durchschnitt verbringen wir 80–90 % unserer Zeit in Innenräumen, meist in der eigenen Wohnung, in Arbeits- und Bildungsstätten oder Freizeiteinrichtungen. Mit jedem Atemzug nehmen wir die Luft des Innenraums zu uns, welche eine Vielfalt an chemischen und biologisch aktiven Stoffen enthalten kann. Zusätzlich wirken sich physikalische Einflüsse wie Temperatur, Luftfeuchtigkeit und Strahlung auf die Raumluft und die Gesundheit aus. Mögliche gesundheitliche Auswirkungen solcher Belastungen können Kopfschmerzen, Müdigkeit, Haut- und Schleimhautreizungen, Allergien sowie Erkrankungen der Lunge und des Herz-Kreislauf-Systems sein. Spätestens seit der COVID-19-Pandemie ist jedem die Bedeutung der Innenraumluft auch für die Übertragung von Krankheitserregern bewusst. In der Regel sind Personen mit Vorerkrankungen besonders anfällig für die Auswirkungen der Innenraumluft.

Wesentliche Einflüsse auf die Raumluftqualität stammen vom Gebäude selbst, etwa in Form gasförmiger Ausdünstungen der verwendeten Baumaterialien oder der Inneneinrichtung. Zugleich beeinflussen wir mit unserem individuellen Lebensstil und der Anwendung verschiedener Produkte (z. B. Reinigungsmittel, Kosmetika) maßgeblich die Beschaffenheit der Innenraumluft. Aber auch menschliche Emissionen wie ausgeatmetes Kohlendioxid (CO_2_) tragen zur Belastung der Luft bei, besonders in stark frequentierten öffentlichen Räumlichkeiten wie Schulen und Bildungseinrichtungen. Feinstaub entsteht durch Aktivitäten wie Backen, Kochen, Kerzenlicht, Kaminfeuer oder die Aufwirbelung von Hausstaub. Biologisch aktive Bestandteile umfassen Hausstaubmilben, Tierallergene, Schimmelpilzsporen und von außen eingebrachte Pollen.

Die Belüftung spielt eine zentrale Rolle bei der Beseitigung von Schadstoffen in Gebäuden, sei es durch Lüftungsanlagen oder das Öffnen von Fenstern. Da einige Luftschadstoffe wie Stickoxide oder Rußpartikel hauptsächlich aus der Außenluft stammen, kann es in besonders belasteten Situationen sinnvoll sein, die Außenluft vor dem Eintritt in das Gebäude zu filtern.

Die Innenraumlufthygiene befasst sich mit den Faktoren, die die Luftqualität in Innenräumen beeinflussen. Sie zielt darauf ab, eine gesundheitsverträgliche Innenraumluft sicherzustellen, idealerweise durch präventive Maßnahmen wie die Auswahl geeigneter Bauprodukte, die Umsetzung von Belüftungskonzepten und die bewusste Nutzung von Konsumprodukten. Lag zu Beginn der Arbeiten der Fokus oft auf der Identifizierung schädlicher Baumaterialien und ihrer Beseitigung, steht mittlerweile die Schaffung eines Bewusstseins für gesundheitsförderliche Bedingungen und die vorausschauende Prävention im Vordergrund.

Die „Innenraumlufthygiene-Kommission“ (IRK) des Umweltbundesamtes (UBA) berät seit knapp 40 Jahren die Bundesregierung, den öffentlichen Dienst und die Fachöffentlichkeit zu zentralen Fragen der Innenraumlufthygiene. Ihre Ergebnisse auf Basis des wissenschaftlichen Erkenntnisstandes münden regelmäßig in Stellungnahmen und Empfehlungen des UBA. Aufgrund der geringen Regulierung der Innenraumluft – es gibt nur einen einzigen gesetzlichen Grenzwert [1] – haben die veröffentlichten Erkenntnisse der IRK in der Regel empfehlenden Charakter und sind rechtlich nicht bindend. Dennoch besitzen sie Vorbildfunktion bei der Lösung der Raumluftprobleme.

Die Empfehlungen der IRK beziehen sich auf sowohl privat als auch öffentlich genutzte Innenräume. Arbeitsstätten im industriellen Sektor, in denen gezielt mit Gefahrstoffen umgegangen wird, werden nicht behandelt, da sie den Bestimmungen des Arbeitsschutzes mit ihrem spezifischen Regelwerk unterliegen. Der Vorsitz der Kommission wird von einer externen Expertin oder einem externen Experten wahrgenommen. Weitere Informationen über die Arbeit der Kommission und ihre Veröffentlichungen sind auf der Webseite der IRK verfügbar.^1^

Gegründet wurde die Kommission 1984 im damaligen zum Bundesgesundheitsamt (BGA) gehörenden Institut für Wasser‑, Boden- und Lufthygiene am Forschungsstandort Berlin-Dahlem. Erklärtes Ziel war es, die zur Innenraumhygiene vorhandenen Kompetenzen in Deutschland zu bündeln und über Ressortgrenzen hinweg Beratung für Bundesregierung und Öffentlichkeit zu gewährleisten.

In den Anfangsjahren ihrer Tätigkeit leistete die IRK Pionierarbeit, indem sie zunächst einen umfassenden Überblick über gesundheitsrelevante Schadstoffe in Innenräumen schuf und anschließend eine Priorisierung der Themenfelder vornahm. Dabei war stets von zentraler Bedeutung, inwiefern die daraus resultierenden Handlungsempfehlungen in der Praxis umsetzbar waren. In den frühen 1980er-Jahren konzentrierten sich die Themen auf anorganische Schadgase, radioaktive Belastungen durch Radon und Thoron sowie Emissionen aus Gasherden, Gas- und Holzheizungen. Daneben waren die Gefahren durch Passivrauchen ein relevantes Thema. Darüber hinaus war es von hoher Dringlichkeit, sich mit den Gefahren auseinanderzusetzen, die durch Wirkstoffe in verwendeten Holzschutzmitteln (zeitweise existierte dafür eine eigenständige „Holzschutzmittel-Kommission“), Schädlingsbekämpfungsmitteln (Pyrethroide), Desinfektionsmitteln und chemischen Reinigungsmitteln entstehen.

Interessanterweise beschäftigte sich die Kommission vom ersten Tag an mit einem Themenkomplex, der nahezu unvermindert relevant geblieben ist, nämlich die flüchtigen organischen Verbindungen (VOC^2^), welche auch über längere Zeiträume aus Baumaterialien, Mobiliar und Konsumprodukten in die Innenraumluft ausgasen. Die besondere Problematik von Formaldehyd war bereits seit den 1970ern bekannt, wenngleich 1984 noch unklar war, inwieweit der Stoff auch krebserzeugend sein kann. (Schon 1977 hatte das BGA einen „Toleranzwert“ für Innenräume von 0,1 ppm abgeleitet.) Forschungsergebnisse zeigten bald, dass man Bauprodukte in Prüfkammern individuell auf raumluftrelevante Ausgasungen prüfen muss und es insbesondere nicht ausreicht, sich auf die zur Verfügung gestellten Rezepturen und Stoffgemische zu stützen. Im Laufe der Zeit wurden standardisierte Prüfverfahren und -bedingungen für die Beurteilung von Emissionen aus Bauprodukten entwickelt, welche heute durch den im Jahr 1997 gegründeten Ausschuss zur gesundheitlichen Bewertung von Bauprodukten (AgBB^3^) umgesetzt werden. Neben der Notwendigkeit der chemisch-analytischen Erfassung der ausgasenden Stoffe wurde auch klar, dass die Gerüche dieser Stoffe eine eigene Art der Wirkung bei den Nutzer:innen von Innenräumen haben. Früh wurde erkannt, dass die öffentlichkeitswirksame Kennzeichnung von Produkten ein maßgeblicher Ansatz zur Verbesserung der Raumluftqualität sein kann. In der IRK wurde daher aktiv daran gearbeitet, brauchbare Anforderungen für die Nutzung des 1978 eingeführten Umweltzeichens „Blauer Engel“ zu formulieren.

Umweltmedizinische und toxikologische Betrachtungen bildeten stets relevante Aufhänger der Arbeiten der IRK: Schon früh finden sich Sick Building Syndrome, allergische Erkrankungen sowie die Toxikologie der Kurz- und Langzeitwirkungen unter den betrachteten Problemen. Rasch war klar, dass man zur gesundheitlichen Beurteilung von Innenraumschadstoffen hygienisch oder toxikologisch begründete Bewertungsmaßstäbe benötigt.

Im Jahr 1990 wurde in der IRK von einem „Bewertungsnotstand“ für Schadstoffe in Innenräumen berichtet, worauf 1994 gemeinsam mit den Bundesländern eine sogenannte Ad-hoc-Arbeitsgruppe ins Leben gerufen wurde. Diese bestand aus Mitgliedern der IRK und der AG-AGLMB Innenraum (Arbeitsgruppe „Innenraumluft“ der Arbeitsgemeinschaft der Leitenden Medizinalbeamten der Länder) und hatte zum Ziel, toxikologisch begründete Richt- bzw. Leitwerte für die Innenraumluft zu entwickeln. Dieses Gremium ist 2015 eine eigenständige Kommission am UBA geworden, die sich aus Vertreter:innen der Länderbehörden, dem Deutschen Institut für Bautechnik (DiBT), dem Institut für Arbeitsschutz der Deutschen Gesetzlichen Unfallversicherung (IFA), dem Bundesinstitut für Risikobewertung (BfR), dem Bundesumweltministerium und dem UBA als Geschäftsstelle zusammensetzt. Heute kennen wir sie als den Ausschuss für Innenraumrichtwerte (AIR; [2]). Die Arbeitsteilung erfolgt so, dass der AIR für die gesundheitliche Bewertung von Innenraumschadstoffen zuständig ist. Hierfür setzt der AIR toxikologisch abgeleitete Richtwerte, aber auch risikobezogene Leitwerte für Kanzerogene sowie hygienische Leitwerte und Geruchsleitwerte fest.^4^ Die IRK bearbeitet die grundsätzlichen praktischen Problemstellungen und Handlungsmaßnahmen zur Innenraumluft. Beide Gremien stehen im laufenden Austausch.

In den 1990er-Jahren richtete sich die Aufmerksamkeit vermehrt auf problematische Stoffe in Gebäuden, die heute als „Altlasten“ bekannt sind. Hierzu gehören PAK (polyzyklische aromatische Kohlenwasserstoffe) aus Asphalt-Fußbodenbelägen und Parkettklebern, PCB (polychlorierte Biphenyle) aus Fugenmassen sowie künstliche Mineralfasern (KMF). Später rückten Asbestanwendungen, insbesondere aus Spachtelmassen, wieder in den Fokus. Angesichts des aktuellen Trends zum nachhaltigen Erhalt von Bausubstanz anstelle von Gebäudeabriss wird die Fachkunde der Mitglieder der IRK immer wieder benötigt, wenn es um die Kenntnis historischer Zusammenhänge und gutachterlicher Befunde bezüglich der Identifikation von Schadstoffen aus der Zeit der Errichtung der Gebäude geht.

Ab den 1990er-Jahren verstärkten sich ebenso die Bemühungen um Energieeinsparung. Gebäude wurden fortan so modernisiert oder von vornherein so konzipiert, dass sie bessere Eigenschaften bezüglich Wärmedämmung und Luftdichtheit aufwiesen. Damit stieg die Bedeutung von gezielten Lüftungsmaßnahmen, um eine hygienisch einwandfreie Innenraumluft zu gewährleisten. Gebäudelüftung spielt eine wesentliche Rolle bei der Abfuhr der entstehenden Stofflasten, sei es vom Gebäude selbst als auch durch die Nutzungsaktivitäten. Die Gewährleistung einer guten Innenraumluft erfordert oft eine Abwägung zwischen der Minimierung der Schadstoffquellen und einer Verbesserung der Belüftungssituation. Das Streben nach einer guten und förderlichen Luftqualität an Schulen begleitet die IRK seit vielen Jahren und dementsprechend hat die Kommission ein umfassendes Kompendium erarbeitet [3].

Ebenso förderte die IRK Bemühungen zur Qualitätssicherung von Innenraummessungen und zur Definition geeigneter Messbedingungen (Ausgleichsbedingungen vs. Nutzungsbedingungen). Ringversuche zur Qualitätssicherung wurden unter Mitwirkung verschiedener Forschungsinstitute und sachverständiger Analyselabore angestoßen.

Neben chemischen Einflüssen gewannen biologische Kontaminationen zunehmend an Bedeutung. Während zu Beginn der 1990er-Jahre die Problematik solcher Kontaminationen in raumlufttechnischen Anlagen akut war, wurde diese bald durch die weitverbreitete Herausforderung von Schimmelpilzen in Gebäuden abgelöst. Durch umfassende Koordinationsarbeit entstanden stark nachgefragte Leitfäden zur Erkennung und Prävention von Schimmelbefall in Gebäuden sowie entsprechende Sanierungsempfehlungen. Als Höhepunkt der Bemühungen wurden 2017 alle Empfehlungen zu Schimmel in einem Gesamtleitfaden zusammengeführt [4].

In den Jahren 2020 bis 2022 erreichte die Diskussion über biologische Kontaminationen mit dem Auftreten des Erregers SARS-CoV‑2 vorläufig ihren Höhepunkt. Während der Pandemiezeit stand die IRK dem UBA fachkundig zur Seite, um brauchbare Empfehlungen für infektionsmindernde Maßnahmen in der Innenraumluft bereitzustellen. Die Kommission förderte den Austausch zwischen den relevanten Behörden und Interessengruppen.

Die jüngste Vergangenheit war geprägt von Diskussionen über neu aufkommende Lifestyle-Produkte wie E‑Zigaretten, Shishas und Ethanolfeuerstellen. Ebenso wurden die Emissionen von Geräten wie Laserdruckern und 3D-Druckern sowie neue Themen wie die potenziellen Auswirkungen von Reithallenstreu oder Mikroplastik behandelt. In diesen Diskussionen erwies sich die Zusammenarbeit mit den Kommissionsmitgliedern aus anderen Bundes- und Länderbehörden, Kommunen und Umweltmessbüros stets als fruchtbar. Zur Bearbeitung spezifischer Themen hat die IRK Fachleute eingeladen, die zeitweise die entsprechenden Arbeitskreise verstärkten.

Im vorliegenden Band finden Sie 2 Stellungnahmen, die während der Berufungsperiode 2022–2025 unter dem Vorsitz von Herrn Frank Kuebart entstanden sind:

- Bauen mit Holz – Empfehlungen für eine gute Raumluftqualität,
- Umnutzung von Gewerbegebäuden in Wohnraum: Was ist bezüglich möglicher Schadstoffbelastungen in der Innenraumluft zu beachten?

Hinzu kommt die Aktualisierung zum bereits genannten Leitfaden zur „Vorbeugung, Erfassung und Sanierung von Schimmelbefall in Gebäuden“ von 2017.

Zu den nach wie vor ungelösten Problemen zählen wir:

- die postpandemische und flächendeckende Gewährleistung einer Basisluftqualität im Innenraum – in Vereinbarkeit mit den Nachhaltigkeitszielen bezüglich Energie und Rohstoffen,
- Gewährleistung guter Raumluftqualität unter den Bedingungen des nachhaltigen Bauens, also unter verstärkter Verwendung von neuen Werkstoffen bzw. Recycling-Bauprodukten,
- eine verbindlichere Produktkennzeichnung – insbesondere auf EU-Ebene – für Bauprodukte unter Berücksichtigung ihrer gesundheitsrelevanten Emissionen in die Innenraumluft,
- Berücksichtigung der Auswirkungen des Klimawandels auf die Raumluftqualität in Gebäuden.

Zusammenfassend halten wir fest: Auf die IRK können Sie zählen, wenn es um Beratung und Empfehlung zu einer gesundheitsförderlichen Innenraumluft geht.

Wir wünschen Ihnen viel Freude bei der Lektüre dieser Ausgabe des Bundesgesundheitsblattes!

### Supplementary Information





## Footnotes

1 Kommission Innenraumlufthygiene, https://www.umweltbundesamt.de/themen/gesundheit/kommissionen-arbeitsgruppen/kommission-innenraumlufthygiene, abgerufen am 17.11.2023.

2 VOC – Volatile Organic Compounds.

3 https://www.umweltbundesamt.de/themen/gesundheit/kommissionen-arbeitsgruppen/ausschuss-zur-gesundheitlichen-bewertung-von, abgerufen am 17.11.2023.

4 https://www.umweltbundesamt.de/themen/gesundheit/kommissionen-arbeitsgruppen/ausschuss-fuer-innenraumrichtwerte, abgerufen am 17.11.2023.
