# Supplementary material for: The air we all breathe: on the work of the Indoor Air Hygiene Commission (IRK)
Source: Bundesgesundheitsblatt Gesundheitsforschung Gesundheitsschutz. 2024 Jan 9;67(1):1–4. [Article in German] doi: 10.1007/s00103-023-03815-z (PMC10776720; doi:10.1007/s00103-023-03815-z)
Supplement: Supplementary file 1 [file 103_2023_3815_MOESM1_ESM.pdf]

This is an English translation of the following article:
Die Luft, die wir alle atmen: Zur Arbeit der Kommission Innenraumlufthygiene (IRK). Editorial. Bundesgesundheitsbl 01 (2024). https://doi.org/10.1007/s00103-023-03815-z

Editorial

The air that we all breathe: on the work of the Indoor Air Hygiene Commission (IRK)

Wolfram Birmili, Anja Daniels, Ana Maria Scutaru
Office of the Indoor Air Hygiene Commission (IRK), Section II 1.3 „Indoor hygiene and health-related environmental impacts“, German Environment Agency, Corrensplatz 1, D-14195, Berlin, Germany. E-Mail: irk@uba.de
Frank Kuebart
Chair of the Indoor Air Hygiene Commission (IRK), eco-Institut Germany GmbH, Schanzenstrasse 6-20, D-51063 Köln, Germany

Dear reader,

on average we spend 80 to 90 per cent of our time indoors, spread among our homes, workplaces, educational establishments and leisure facilities. With every breath we take, we inhale indoor air that can contain a variety of chemical and biologically active substances. In addition, physical influences such as temperature, humidity and radiation can have effects on indoor air, comfort and health. Possible health effects of indoor pollution can include headaches, fatigue, skin and mucous membrane irritation, allergies and diseases of the lung and cardiovascular system. Since the COVID-19 pandemic, at the latest, everyone has been aware of the importance of indoor air for the transmission of infectious pathogens. As a general rule, people with pre-existing illnesses tend to be more susceptible to the effects of indoor air.

Significant influences on indoor air quality originate from the building itself, for example in the form of gaseous vapours from the building materials or interior fittings used. At the same time, our individual lifestyle and the use of associated products such as cleaning agents or cosmetics have a significant influence on the quality of indoor air. However, human emissions such as exhaled carbon dioxide (CO_2) also contribute to indoor air pollution, especially in densely occupied public spaces such as schools and educational establishments. Fine particulate matter is created by activities such as baking, cooking, candlelight, open fires, or the resuspension of house dust. Biologically active components include house dust mites, animal-derived allergenes, mould spores and pollen brought in from ambient air.

Ventilation plays a key role in the removal of pollutants from buildings, whether through mechanical ventilation (HVAC) systems or by simply using windows. Some air pollutants such as nitrogen oxides or soot particles originate mainly from ambient air, so it can be useful in some situations to filter the ambient air when it enters the building.

Indoor air hygiene deals with the factors that influence indoor air quality. It aims to ensure healthy indoor air, ideally through preventative measures such as the selection of suitable building products, the implementation of ventilation concepts and the conscious application of consumer products. While early work in indoor hygiene was often concerned with identifying harmful building materials and their removal, emphasis has now changed towards creating higher awareness of health-promoting conditions and prospective prevention.

The Indoor Air Hygiene Commission (IRK) of the Federal Environment Agency (UBA) has been advising the Federal Government, the public service sector and the expert public on key issues of indoor air hygiene for almost 40 years. Its findings are based on scientific knowledge and regularly result in UBA statements and recommendations. Due to the low level of regulation of indoor air - there is just one single legal limit value in Germany[1] - the IRK's publications are generally of a recommendatory nature and not legally binding. Nevertheless, they serve as an important model for solving the occurring indoor air problems.

The recommendations of IRK relate to both private and public indoor spaces. Workplaces in the industrial sector, in which hazardous substances are handled on a regular basis, are usually not considered as they are subject to the specific regulations of occupational health and safety at workplaces. The Indoor Air Hygiene Commission is chaired by an external expert. For detailed information on the work of the Commission and its publications visit IRK's website[2].

The commission was founded in 1984 at the Institute for Water, Soil and Air Hygiene, which was then part of Germany's Federal Health Office (BGA) located in West Berlin. IRK's mission was to pool the existing expertise on indoor hygiene in Germany and provide advice to the federal government and the public across departmental boundaries.

In the early years IRK carried out pioneering activities by creating a comprehensive overview of health-relevant pollutants in indoor spaces and then prioritising the topics. The extent to which its resulting recommendations and guidelines could be implemented in practice was always of central importance. In the early 1980s, the focus was on harmful inorganic gases including radioactive contamination from radon and thoron as well as emissions from gas cookers, gas and wood-fired heating systems. The risks of passive smoking in indoor spaces were also addressed. In addition, it was a matter of great urgency to address the dangers posed by harmful substances in wood preservatives (in the era there was even a separate "Wood Preservatives Commission"), pesticides (pyrethroids), disinfectants and chemical cleaning agents used in indoor environments.

From the very first day, the Commission was concerned with a complex of topics that has remained almost as relevant as ever, namely volatile organic compounds (VOCs), which are emitted from building materials, furniture and consumer products into indoor air. The particular issue of formaldehyde contaminations had been known since the 1970s, although in 1984 it was still unclear to what extent the substance could also be carcinogenic. (Germany's Federal Health Office had already issued a "tolerance value" of 0.1 ppm for

---

[1]Richtwerte für Tetrachlorethen in der Innenraumluft. Bundesgesundheitsbl. 60, 1305–1315 (2017). https://doi.org/10.1007/s00103-017-2637-3

[2] Indoor Air Hygiene Commission (IRK- Kommission Innenraumhygiene), https://www.umweltbundesamt.de/en/topics/health/commissions-working-groups/indoor-air-hygiene-commission, access on Nov 17, 2023.

indoor spaces in 1977). Research soon showed that building products had to be tested individually in test chambers for outgassing substances and that it was not sufficient to rely on the formulations and mixtures provided for construction materials. Over time, standardised test methods and conditions were developed for the assessment of emissions from building products, which are now systematically implemented by the Committee for Health-related Evaluation of Building Products (AgBB[3]) founded in 1997. In addition to the need for chemical analysis of outgassing substances, it also became evident that the odours of these substances have their own specific effects on the users of indoor spaces. It was recognised early on that publicly effective labelling of products could be a significant approach to improving indoor air quality. IRK helped towards formulating useful requirements for the use of the "Blue Angel" eco-label introduced earlier in 1978.

Environmental medical and toxicological considerations have always been a main driver for IRK’s work, ranging from the "sick building syndrome", allergic diseases to the toxicology of short and long-term effects. It soon became clear that systematically derived assessment standards – based on hygienic or toxicological evidence – were needed for the health assessment of indoor pollutants. In 1990 IRK reported an " emergency situation" for the assessment of indoor pollutants, whereupon the so-called "ad hoc working group" was set up in 1994 in cooperation with the German federal states. This working group consisted of members of the IRK and the AG-AGLMB (Working Group "Indoor Air" of the Working Group of the Chief Medical Officers of the Federal States) and aimed to develop toxicologically based guideline values for indoor air. In 2015, this committee became an independent commission at the UBA, made up of representatives of the state authorities, the German Institute for Building Technology (DiBT), the Institute for Occupational Safety and Health of the German Social Accident Insurance (IFA), the Federal Institute for Risk Assessment (BfR), the Federal Ministry for the Environment and the UBA as the office. Today, we know it as the Committee for Indoor Air Guide Values (AIR)[4]. The division of tasks is such that AIR is responsible for the health assessment of indoor pollutants[5], sets toxicologically-derived guide values, risk-related guide values for carcinogens, hygiene guide values and odour guide values. The IRK, on the other hand, deals with the fundamental practical problems and measures relating to indoor air. Both committees are in constant dialogue.

In the 1990s, attention was directed on problematic substances in buildings, which are now known as "contaminated sites". Inherited pollutants include PAHs (polycyclic aromatic hydrocarbons) from asphalt floor coverings and parquet adhesives, PCBs (polychlorinated biphenyls) from sealing materials as well as man-made mineral fibres. Later, asbestos applications, particularly from levelling compounds (filler), came back into focus. In view of the current trend towards the sustainable preservation of building fabric instead of building demolition, the expertise of the members of the IRK is required when it comes to knowledge of historical contexts and expert findings regarding the identification of pollutants from the time the buildings were constructed.

The 1990s also saw increasing efforts to save heating energy in buildings. Consequently, buildings have been retrofitted or designed from the outset in such a way that they had

---

[3] https://www.umweltbundesamt.de/en/topics/health/commissions-working-groups/committee-for-health-related-evaluation-of-building, access on Nov 17, 2023.

[4] Martin Kraft (2020): Atmen Sie jetzt tief ein! Von der Arbeit des Ausschusses für Innenraumrichtwerte (AIR) Bundesgesundheitsbl, 63:1187–1188, https://doi.org/10.1007/s00103-020-03219-3

[5] https://www.umweltbundesamt.de/themen/gesundheit/kommissionen-arbeitsgruppen/ausschuss-fuer-innenraumrichtwerte, access on Nov 17, 2023.

better thermal insulation and airtightness. This enhanced the importance of targeted ventilation measures to ensure hygienically perfect indoor air. Building ventilation plays an essential role in the removal of the material loads generated, both by the building itself and by the activities in which it is used, and ensuring good indoor air often requires a trade-off between minimising the sources of pollutants and improving the ventilation situation. The pursuit of good and favourable air quality in schools has accompanied the IRK for many years and the commission has compiled a comprehensive compendium accordingly[6].

IRK also promoted efforts to ensure the quality of indoor measurements and to define suitable measurement conditions (pseudo-equilibrium conditions vs. conditions of use). Interlaboratory tests for quality assurance were fostered in cooperation with various research institutes and expert analysis laboratories.

In addition to chemical influences, biological contaminations became increasingly relevant. While the problems of contaminations in ventilation systems was acute at the beginning of the 1990s, this was soon superseded by the widespread challenge of mould in buildings. Extensive coordination work led to the creation of popular guidelines for the detection and prevention of mould infestation in buildings as well as corresponding remediation recommendations. As a culmination of these efforts, all recommendations on mould were finally merged into an overall guideline in 2017[7].

During 2020-2022, the discussions on biological contaminations reached their peak for the time being with the spread of the SARS-CoV-2 pathogen. During the pandemic, IRK provided expert support to the UBA in order to provide useful recommendations for infection-reducing actions in indoor air. The Commission promoted dialogue between the relevant authorities and interest groups.

During the recent years, IRK activities have revolved around emerging lifestyle products such as e-cigarettes, shishas and ethanol fireplaces. Emissions from devices such as laser printers and 3D printers as well as new topics such as the potential impact of indoor riding arena bedding as well as microplastics were also discussed. During these discussions, cooperation with the commission members from other federal and state authorities, municipalities, and environmental measurement companies always proved fruitful. IRK regularly invites experts to collaborate on specific topics.

In this volume you find two statements that were produced during the 2022-2025 appointment period under the chairmanship of Frank Kuebart:

- Building with wood - recommendations for good indoor air quality
- Conversion of industrial buildings into housing space: What needs to be considered with regard to possible pollutants in the indoor air?

In addition, there is an amendment to the aforementioned “Guideline on the prevention, detection and remediation of mould in buildings” from 2017.

---

[6] UBA (2008) Leitfaden für die Innenraumhygiene in Schulgebäuden, 142 S. https://www.umweltbundesamt.de/sites/default/files/medien/publikation/long/3689.pdf, access on Nov 1, 2023.

[7] UBA (2017) Guideline on the prevention, detection and remediation of mould in buildings. 192 p. https://www.umweltbundesamt.de/publikationen/guideline-mould, access on Nov 1, 2023.

Challenges that remain unresolved include:

- Ensuring a basic indoor air quality in the post-pandemic age - in line with sustainability targets for energy and raw materials.
- Ensuring a good indoor air quality under the conditions of sustainable building, i.e. under an increased use of new materials and/or recycling building products
- More stringent and binding product labelling - particularly at EU level - for construction products, taking into account their health-related emissions into indoor air
- Anticipating the effects of climate change on indoor air quality in buildings

To sum up: you can count on our Indoor Air Hygiene Commission for advice and recommendations on healthy indoor air!

We hope you enjoy reading this special issue!

Conflict of interest
Wolfram Birmili, Anja Daniels, Ana Maria Scutaru, Frank Kuebart declare no conflict of interest.
